# TGF-β inducible epithelial-to-mesenchymal transition in renal cell carcinoma

**DOI:** 10.18632/oncotarget.26682

**Published:** 2019-02-19

**Authors:** Sandy Tretbar, Peter Krausbeck, Anja Müller, Michael Friedrich, Christoforos Vaxevanis, Juergen Bukur, Simon Jasinski-Bergner, Barbara Seliger

**Affiliations:** ^1^ Martin Luther University Halle-Wittenberg, Institute for Medical Immunology, 06112 Halle, Germany; ^2^ State Hospital, Healthcare Centre Glantal, 55590 Meisenheim, Germany

**Keywords:** epithelial-to-mesenchymal transition, renal cell carcinoma, TGF-β, Smad-signaling pathway, inhibition

## Abstract

Epithelial-to-mesenchymal transition (EMT) is a crucial step in cancer progression and the number one reason for poor prognosis and worse overall survival of patients. Although this essential process has been widely studied in many solid tumors as e.g. melanoma and breast cancer, more detailed research in renal cell carcinoma (RCC) is required, especially for the major EMT-inducer transforming growth factor beta (TGF-β). Here, we provide a study of six different RCC cell lines of two different RCC subtypes and their response to recombinant TGF-β1 treatment. We established a model system shifting the cells to a mesenchymal cell type without losing their mesenchymal character even in the absence of the external stimulus. This model system forms a solid basis for future studies of the EMT process in RCCs to better understand the molecular basis of this process responsible for cancer progression.

## INTRODUCTION

Renal cell carcinoma (RCC) is among the ten most frequent forms of cancer with still poor prognosis [1] and can be histologically classified into 3 major subgroups: clear cell type as the most frequent form of RCC (ccRCC, 75–85%), papillary (pRCC, 13–15%) and chromophobe type (chRCC, 5%) [2]. The ccRCC type is often characterized by aberrations in the *VHL* gene on chromosome 3p, usually causing the loss of the VHL-mediated degradation of the hypoxia-inducible factor alpha (HIF-α) under normoxic conditions [3, 4]. This leads to a metabolic switch to aerobic glycolysis [5, 6] and drastic changes in the composition of the tumor microenvironment (TME) associated with impaired immune recognition of the tumor by immune cells [7–9]. The pRCC has an aggressive, highly lethal phenotype and is divided in type 1 and 2 based on histological staining and specific genetic alterations [2, 10]. The chRCC subtype demonstrates a low rate of somatic mutation compared to most tumors and carries the best prognosis among RCCs [2, 11]. Together the three main subgroups represent more than 90% of all RCCs [2, 12].

About 30% of the tumors are already metastatic at initial diagnosis and 30–40% of the patients develop metastasis after initial nephrectomy [13]. The underlying process driving cancer progression, aggressiveness and metastasis is the epithelial-to-mesenchymal transition (EMT) of tumor cells. This process is associated with an altered expression of cell surface markers, transcription factors (TF), microRNAs (miRNAs), cytoskeletal proteins, extracellular matrix (ECM) components, and cell surface markers [14]. EMT can be induced by a number of growth factors [15] binding to their cognate receptor leading to signal cascades that either directly affect epithelial properties or regulate downstream processes via TFs [15]. The hallmark of EMT is the repression of E-cadherin by Zinc finger E-box-binding homeobox 1 (ZEB1) and Snail TF-family members and induction of matrix metalloproteases (MMP) resulting in enhanced motility/plasticity, invasiveness as well as increased resistance to apoptosis of tumor cells [16–18].

In general, elevated levels of cytokines and chemokines were shown to drive tumor progression and aggression in RCC [19]. The tumor necrosis factor alpha (TNF-α) and the cytokine interleukin 15 (IL-15) are experimentally proven inducers of EMT in RCC [20, 21]. High levels of the transforming growth factor beta (TGF-β) expression were found in RCC cells in comparison to normal kidney epithelium [19]. Furthermore, increased levels of TGF-β1 and TGF-β signaling were associated with the loss of epithelial differentiation [22]. TGF-β1 can exert its function via the canonical (Smad-dependent) and non-canonical (Smad-independent) signaling pathway. In the canonical pathway, TGF-β1 binds to its cognate TGF-β receptor type II (TGFBR2) leading to receptor activation and heterotetramer formation with the type I receptor dimer (TGFBR1). The kinase domain of TGFBR2 phosphorylates the TGFBR1 subunit resulting in Smad2/3 phosphorylation by TGFBR1, association of Smad2/3 with Smad4 and transfer to the nucleus. There, the Smad2/3-Smad4 complex associates with DNA binding partners in order to repress or enhance transcription of downstream targets [23–25]. In ccRCC, the TGF-β/Smad signaling pathway was shown to drive tumor progression and invasiveness [19]. Downstream targets of this pathway are MMP2 and MMP9 and high expression levels of these two proteinases directly correlate with poor prognosis in RCC [26]. Upregulation of Snail promotes tumor metastasis in RCC *in vitro* and *in vivo* [27] and is significantly associated with tumor grading and staging as well as with the presence of sarcomatoid differentiation [28].

Although TGF-β1 is one of the most well-known inducers for EMT and the TGF-β/Smad-signaling pathway is well studied for a variety of solid tumors [29–33], the TGF-β1 driven EMT in RCC is still poorly understood. Therefore, we studied the effect of TGF-β1 treatment on growth properties, phenotype, and gene expression pattern in the two most common RCC subtypes ccRCC and pRCC by characterization of their ability to transition from an epithelial to a mesenchymal cell type using microscopy, flow cytometry, qRT-PCR and Western blot analysis, respectively. Since changes in the immunogenicity of tumor cells were postulated during EMT [34], the effect of TGF-β1 treatment on immune modulatory molecules, such as major histocompatibility complex class (MHC) I surface antigens and co-stimulatory/inhibitory molecules, was studied using flow cytometry and qRT-PCR. In addition, the reversibility of this transition process and its underlying mechanism were investigated using re-culturing and inhibition experiments. Our study supports an irreversible transition of RCC cells to a mesenchymal cell type once they were stimulated with external recombinant TGF-β1 protein. Furthermore, we provide a model for a self-enforcing feedback-loop that keeps up the mesenchymal cell type even when the external stimulus was removed from the system.

## RESULTS

### The effect of TGF-β1 treatment on cell properties and morphology

To test whether exogenous TGF-β1 treatment has an effect on survival and growth, five ccRCC cell lines (786-O, Caki-1, Caki-2, MZ1851RC, MZ2733RC) and one pRCC cell line (MZ2858RC) were left untreated or treated with 10 ng/mL TGF-β for 48 to 96 hours, before their cell viability, proliferation and apoptosis was analyzed. Cell viability and proliferation of the RCC cell lines was comparable over a period of 96 h and upon TGF-β1 treatment. Additionally, the apoptosis rate of RCC cells was not enhanced in the presence of TGF-β1 demonstrating that the treatment of the RCC cells with exogenous TGF-β1 does not interfere with their growth properties and does not lead to apoptosis or necrosis with their survival (Supplementary Figure 1).

**Figure 1:**
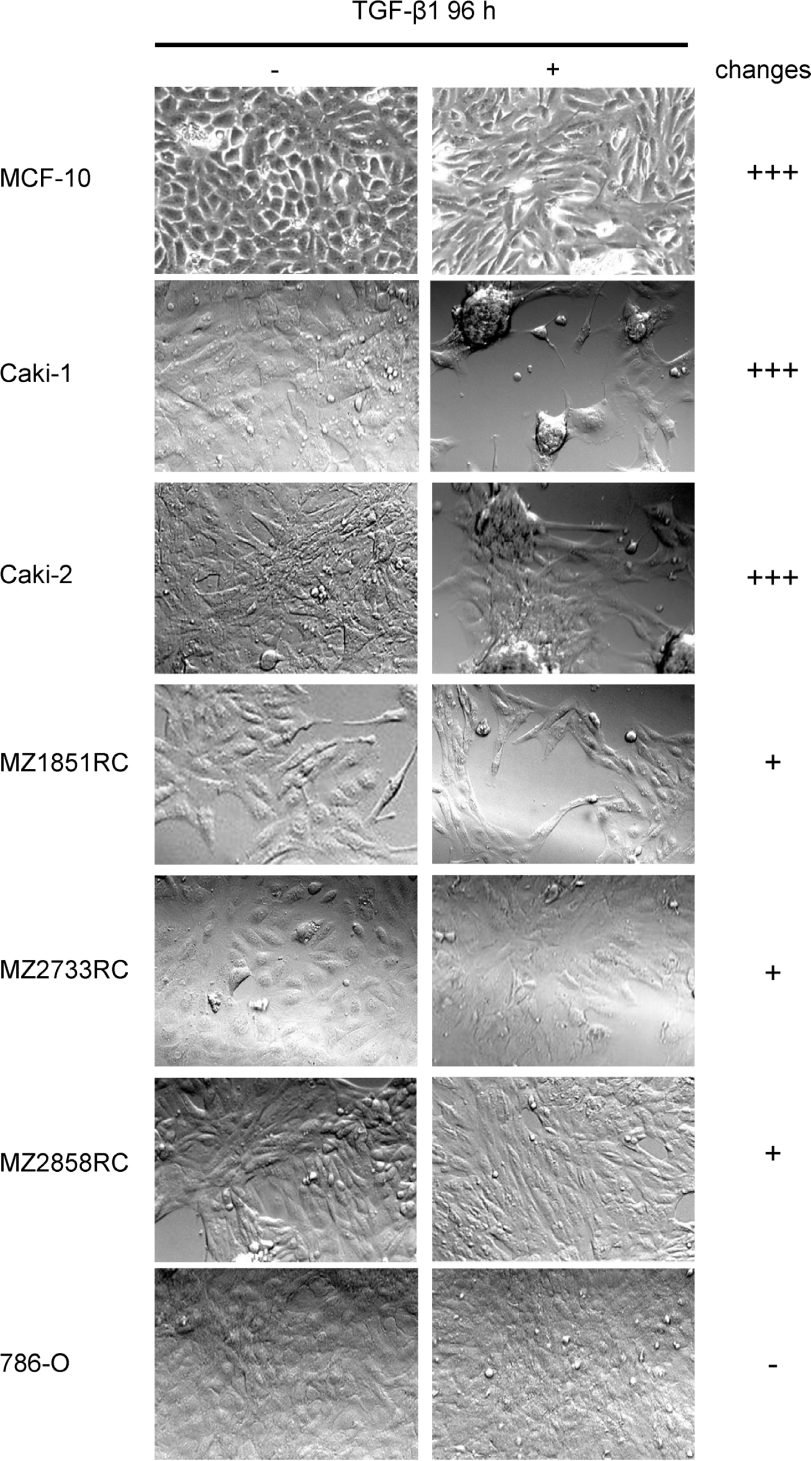
Morphology changes of RCC cell lines and MCF-10 after TGF-β1 treatment Cells were treated for 96 h with 10 ng/ml TGF-β or left untreated and morphological changes were monitored by microscopy. Representative photos of at least 3 independent experiments are shown.

In order to establish a TGF-β-inducible EMT system for RCC cell lines, the mammary gland cell line MCF-10 served as a prototype, since MCF-10 cells properly transition from epithelial to mesenchymal cells upon TGF-β1 treatment [30]. MCF-10 cells undergoing EMT typically have been shown to lose their apical-basolateral polarity and acquire a more fibroblast-like shape [17, 35]. Therefore, along with MCF-10, the 6 different RCC cell lines were analyzed for morphological changes upon TGF-β1 treatment using light microscopy. As shown in Figure 1, heterogeneous results were obtained for the 7 different cell lines, which could be classified into 3 groups according to their extent of morphological change: MCF-10, Caki-1, and Caki-2 showed drastic changes in morphology upon TGF-β1 stimulation (+++); MZ1851RC, MZ2733RC, and MZ2858RC showed minor differences after TGF-β1 stimulation (+) while for 786-O no obvious differences in shape were detected after TGF-β1 treatment (-) (Figure 1).

### Functional TGF-β/Smad signaling pathway in RCC cell lines

**Figure 2:**
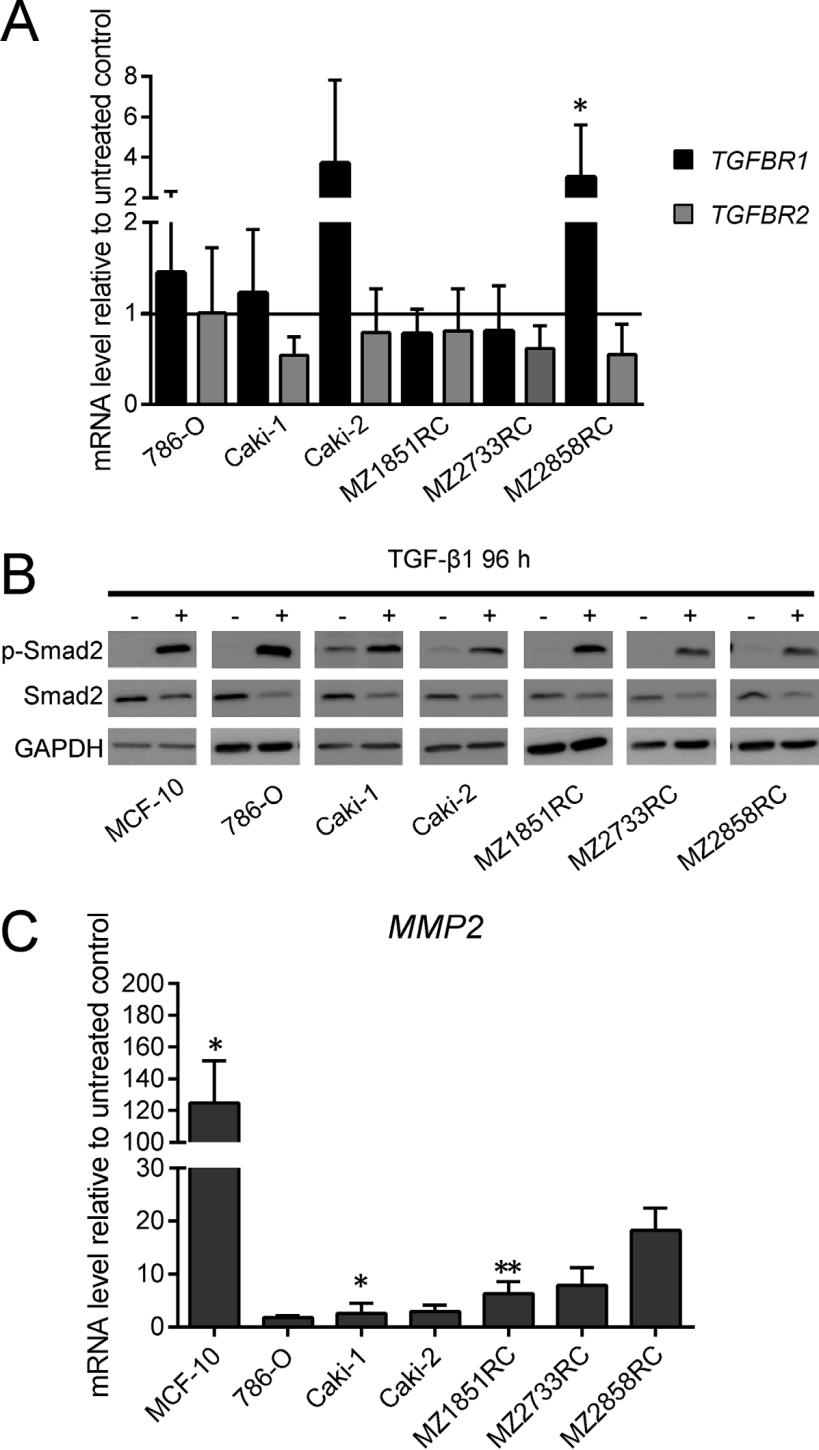
Effect of TGF-β1 treatment on key players of the TGF-β/Smad-signaling pathway All bar graphs show mRNA levels determined by qPCR relative to the unstimulated control and represent mean values of at least 3 biological replicates (*n* ≥ 3). (**A**) The mRNA levels of *TGFBR1* and *2* relative to the unstimulated control are shown for *TGFBR1* as black bars, for *TGFBR2* as grey bars. (**B**) One representative out of 3 biological replicates of Western blots is shown (*n* = 3). According to the loading control GAPDH, the signal intensity for phosphorylated Smad2 is more abundant after TGF-β1 treatment, while the overall protein level of Smad2 seems reduced after TGF-β1 treatment. (**C**) *MMP2* mRNA levels are increased upon TGF-β1 treatment. (^*^p ≤ 0.05, ^**^p ≤ 0.01).

As a prerequisite for analyses of the effect of exogenous TGF-β1 on the TGF-β/Smad signaling pathway, the expression of TGF-β and its receptors was determined by qPCR. All RCC cell lines constitutively expressed TGF-β1, TGFBR1 and TGFBR2. After treatment with TGF-β1, the mRNA level of *TGFBR2* was down-regulated in all RCC cells, while *TGFBR1* levels were rather increased or not regulated in these cells with the exception of MZ2733RC, in which a down-regulation of *TGFBR1* mRNA levels was detected (Figure 2A). Monitoring the response of the tumor cells to TGF-β1 by analyzing the phosphorylation status of Smad2 demonstrated an increased phosphorylation status of Smad2 upon TGF-β1 treatment, but the overall amount of protein remained the same in all RCC cell lines tested (Figure 2B). Furthermore, the transcription of *MMP2*, a downstream target of the TGF-β-inducible Smad signaling pathway, was 2- to 100-fold upregulated in treated cells (Figure 2C). Both Western blot and qPCR data indicate a functional TGF-β/Smad signaling pathway in the 6 RCC cell lines analyzed, which was comparable to that of the well-studied MCF-10 cell line.

### TGF-β-inducible EMT in RCC cell lines

**Figure 3:**
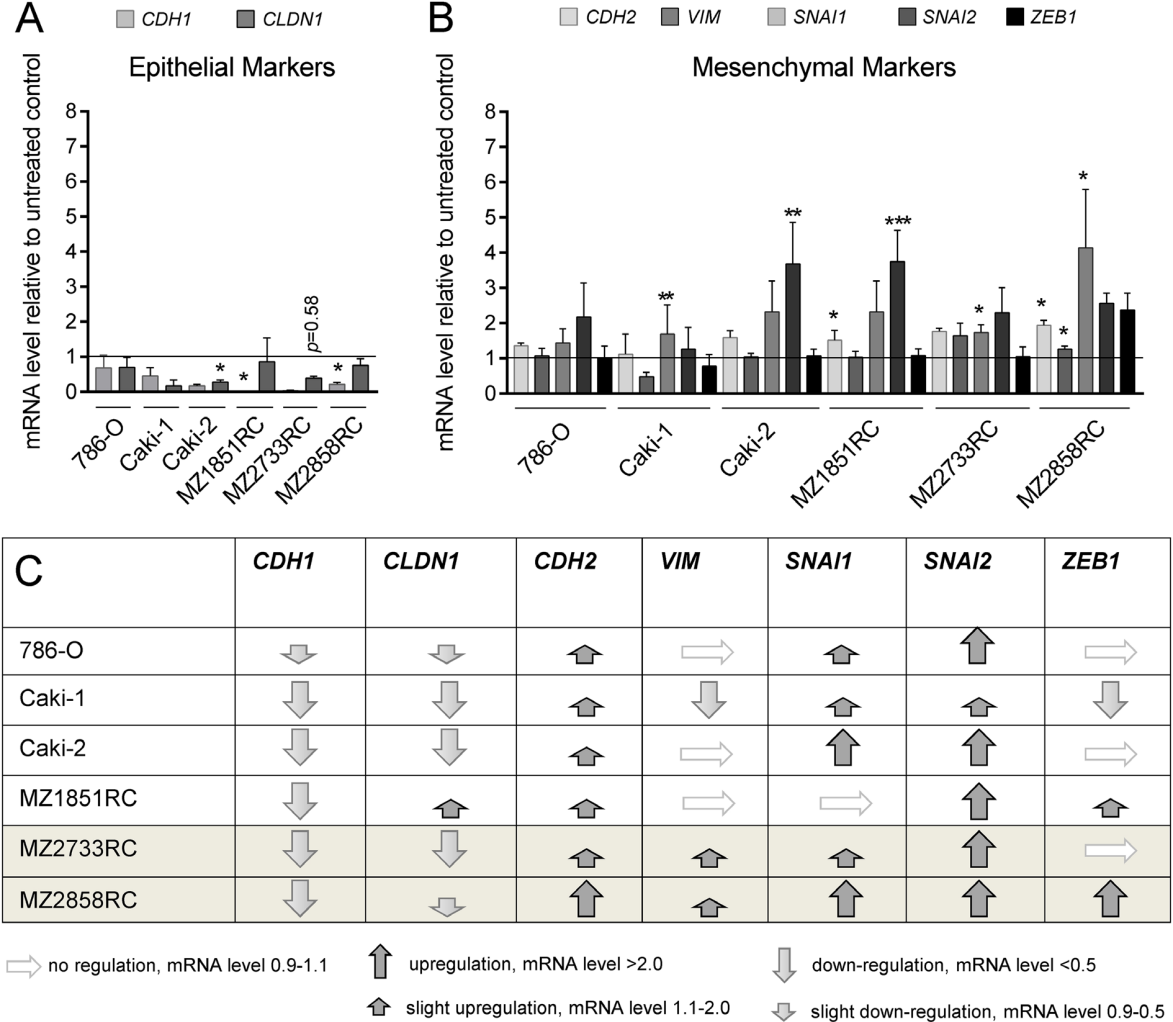
TGF-β1 treatment stimulates EMT in RCCs All bar graphs show mRNA levels determined by qPCR relative to the unstimulated control and represent mean values of at least 3 biological replicates (*n* ≥ 3). (**A**) The epithelial markers *CDH1* and *CLDN1* were reduced after TGF-β1 treatment in all RCC cell lines tested. (**B**) In general, mesenchymal markers were either not regulated or upregulated after TGF-β1 treatment in six different RCC cell lines. For Caki-1, a down-regulation of *VIM* and *ZEB1* was observed. (**C**) Table summarizing the regulation of epithelial and mesenchymal marker expression in seven different RCC cell lines. Grey shaded rows indicate the ccRCC (MZ2733RC) and the pRCC cell line (MZ2858RC) responding best to the TGF-β1 treatment. (^*^p ≤ 0.05, ^**^p ≤ 0.01, ^***^p ≤ 0.001).

Since the TGF-β/Smad-signaling pathway is functional in the RCC cell lines, the expression of EMT markers was analyzed in the presence and absence of TGF-β1. In general, the expression of the epithelial markers E-cadherin, cytokeratins, occludins, and claudins decrease during the transition process, whereas TFs like Snail (SNAI1), Slug (SNAI2), ZEB1, ZEB2, and Twist as well as mesenchymal markers such as N-cadherin, vimentin, MMPs, and fibronectin are upregulated [14, 17, 36–38]. Selected EMT markers were investigated at the mRNA level via qPCR. All biomarkers analyzed followed the typical pattern for EMT: *CDH1* and *CLDN1* were down-regulated after TGF-β1 treatment (Figure 3A), whereas *SNAI1*, *SNAI2*, *ZEB1*, *CDH2* and *VIM* were mainly upregulated (Figure 3B). Interestingly, all 6 RCC cell lines underwent EMT to a certain extent upon stimulation with TGF-β1. However, the ccRCC cell line MZ2733RC and the pRCC cell line MZ2858RC responded best to the TGF-β1 stimulus (Figure 3C) and were selected for further investigations.

### Improved cell mobility upon TGF-β1 treatment

**Figure 4:**
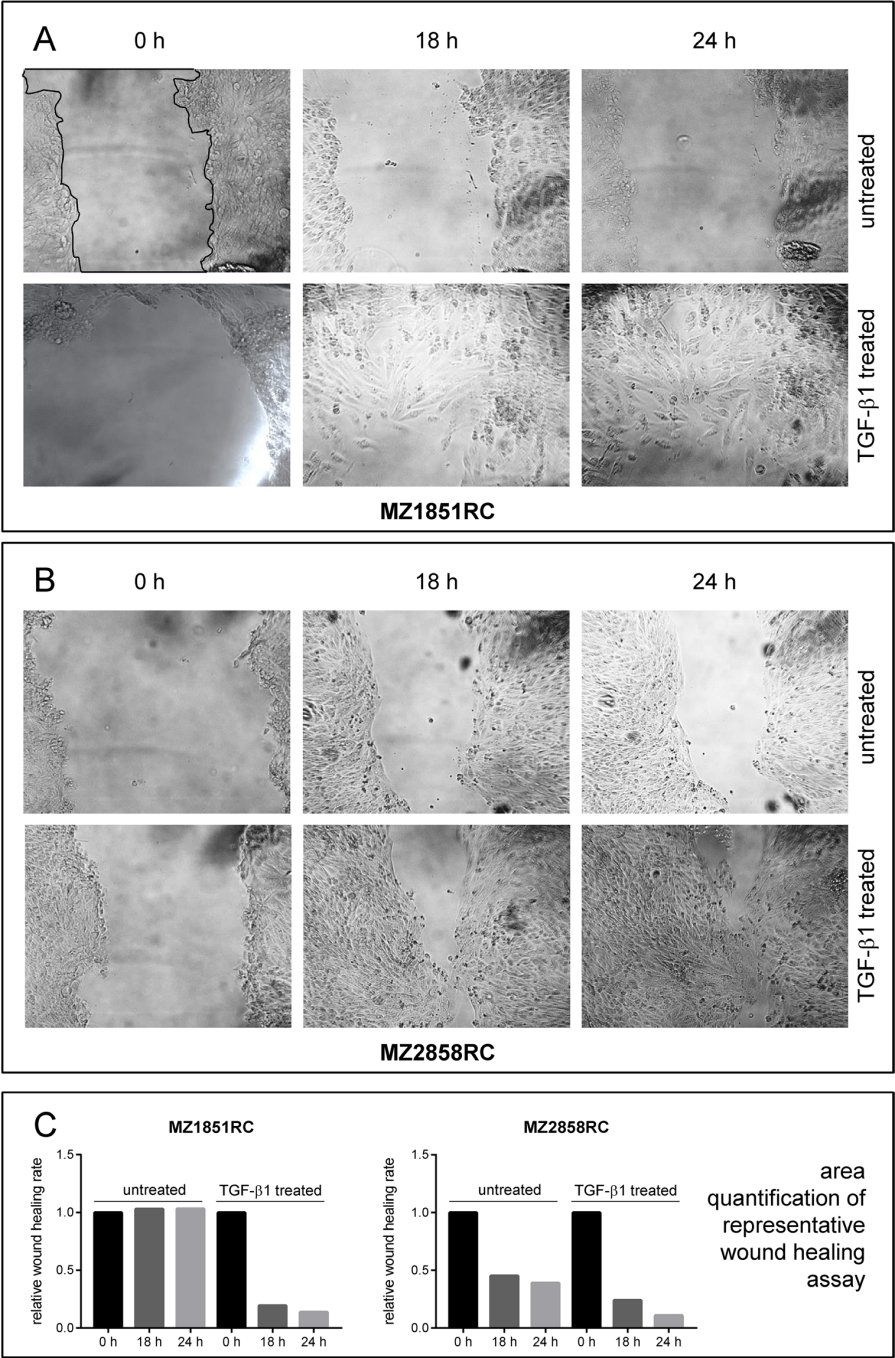
Wound healing properties of RCC cell lines after TGF-β1 treatment Cells were either left untreated or treated for 96 h with 10 ng/ml TGF-β and the ability of wound healing was monitored by microscopy. Representative imagess of at least 3 independent experiments are shown for the ccRCC cell line MZ1851RC (**A**) and the pRCC cell line MZ2858RC (**B**). The area of the scratch was quantified using ImageJ and displayed as bar graph relative to the 0 h time point (**C**). A representative line for scratch area quantification (black line) is shown in (**A**), upper left panel.

Due to the clear cell phenotype, the morphology of MZ2733RC was only hardly detected by light microscopy. Therefore, the ccRCC cell line MZ1851RC and the pRCC cell line MZ2858RC were representative cell lines chosen for wound healing assays. In the presence of TGF-β1, the wound healing was improved for both cell lines due to increased cell mobility (Figure 4) and this process was nearly completed after 24 h of setting the scratch.

### Effect of TGF-β1 treatment on immune modulatory molecules in RCC

**Figure 5:**
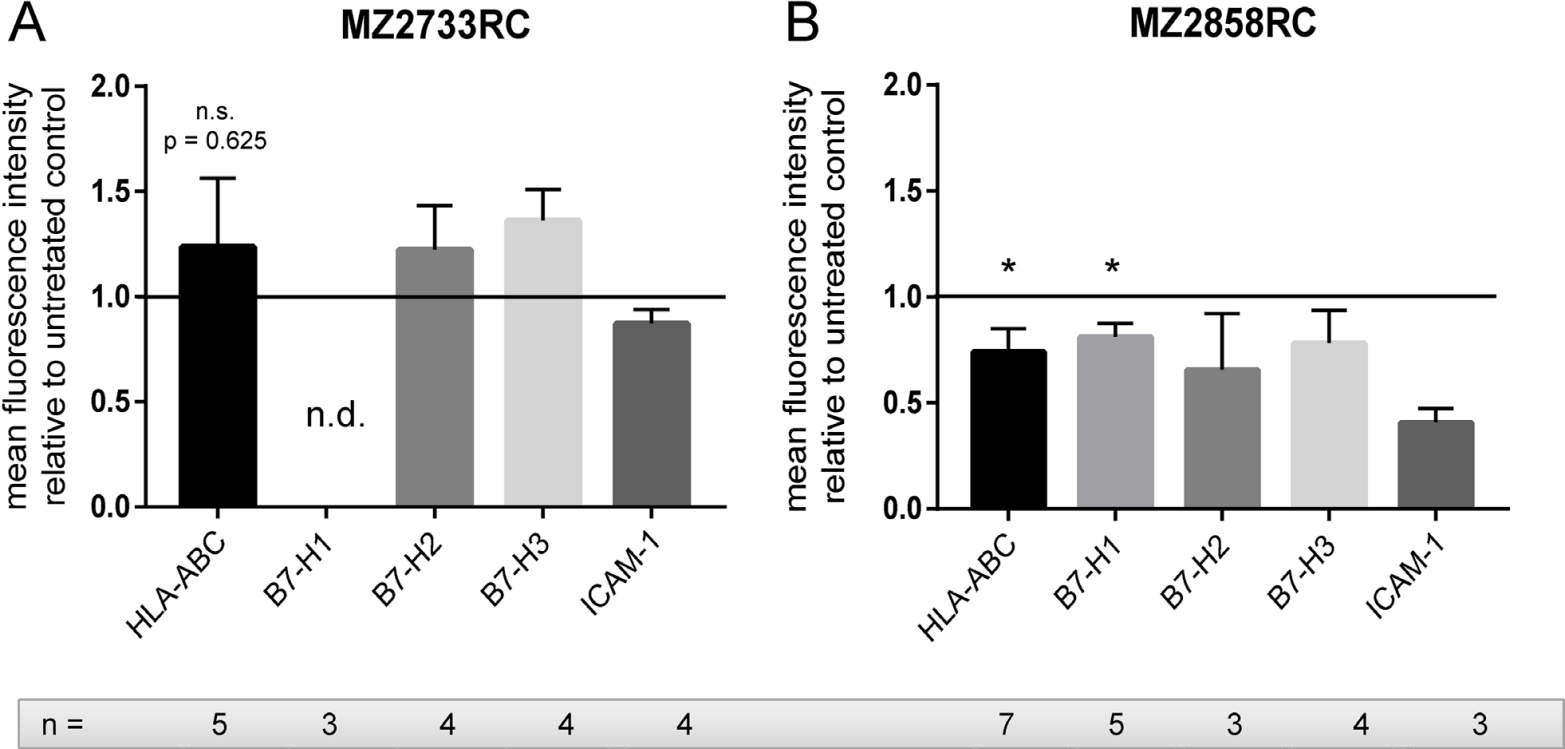
The effect of the TGF-β1 treatment on the expression of immune modulatory cell surface molecules Bar graphs show flow cytometry data of at least 3 independent stimulation experiments (*n* ≥ 3) normalized to the untreated control. (**A**) HLA-ABC, B7-H2, and B7-H3 are upregulated, whereas HLA-BC and ICAM-1 are down-regulated after TGF-β1 treatment in MZ2733RC. (**B**) In MZ2858RC, all tested proteins are down-regulated after TGF-β1 treatment. (n.s.: not significant, ^*^p ≤ 0.05, n.d.: not detected).

RCC is often characterized by the down-regulation of the HLA class I surface expression and aberrant expression of components of the antigen presenting and processing machinery (APM, [39]) and co-inhibitory molecules. Therefore, the effect of the TGF-β1 treatment on expression of HLA class I antigens and co-stimulatory molecules was analyzed in the ccRCC cell line MZ2733RC and the pRCC cell line MZ2858RC by flow cytometry and qPCR. Using antibodies directed against HLA-ABC, B7-H1 (PD-L1), B7-H2, B7-H3, and ICAM-1, the surface protein expression in both cell lines was monitored prior and after TGF-β1 treatment. As shown in Figure 5, a heterogeneous TGF-β1-regulated expression pattern was found in both cell lines.

In MZ2858RC, HLA-ABC, B7-H1, B7-H2, B7-H3, and ICAM-1 were down-regulated after TGF-β1 stimulation. In contrast, HLA-ABC, B7-H2, and B7-H3 were upregulated in the ccRCC MZ2733RC. Comparable to MZ2858RC, the expression of the cell adhesion molecule ICAM-1 was reduced after TGF-β1 treatment of MZ2733RC. No surface expression of B7-H1 was observed for MZ2733RC. Analysis of APM components on mRNA level demonstrated mostly a down-regulation of the four genes tested after TGF-β1 treatment in both cell lines (Supplementary Figure 1) which is in accordance with MHC I surface expression in MZ2858RC, but not in MZ2733RC.

### Reversibility of the mesenchymal transition in RCC cells

**Figure 6:**
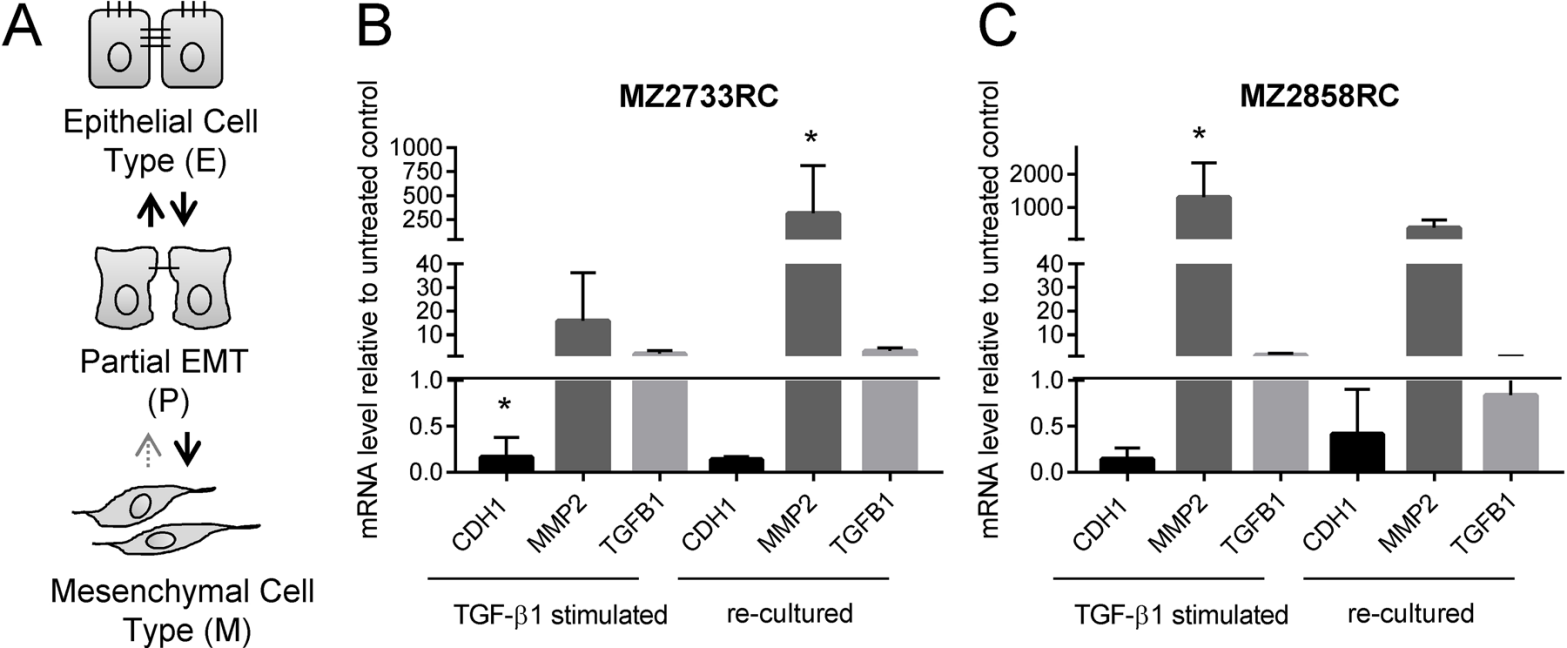
Reversibility of the mesenchymal transition of representative RCC cell lines analyzed by qPCR (**A**) Schematic representation of EMT states. (**B–C**) Bar graphs show data of 3 independent re-culturing experiments. Black bars display the mRNA levels of the epithelial marker *CDH1*. Dark grey bars indicate the mRNA levels of the mesenchymal marker *CDH2*; the light grey bars show the mRNA levels of TGF-β1 (*TGFB1*). All values are displayed relative to the untreated control after 96 h of TGF-β1 treatment or additional 96 h re-culturing, respectively. (^*^p ≤ 0.05).

Different amounts of TGF-β can lead to a different EMT transition status in the cells suggesting a concentration-dependent EMT process. In general, epithelial (E) cells transition to a mesenchymal (M) cell type via a metastable intermediate state, known as partial (P) EMT [30, 40–42]. Transitions from E to P states are reversible [30, 42], while formation of the M state is mostly irreversible [30] (Figure 6A). Since no remarkable changes of EMT marker patterns were detected with increasing amounts of TGF-β1 (10–100 ng/mL, Supplementary Figure 2A), the RCC cell lines were investigated whether they partially or irreversibly transit to the mesenchymal cell type with 10 ng/mL TGF-β1 within a time span of 96 h (a longer time did not considerably increase the response to the TGF-β1 stimulus, see Supplementary Figure 2B).

To study the reversibility of the mesenchymal transition, 5 ccRCC and 1 pRCC cell line were treated with TGF-β1, re-cultured in medium in the absence of TGF-β1 and subsequently analyzed for EMT markers at the mRNA level (Figure 6B, Supplementary Figure 3). Analysis at the protein level via flow cytometry according to Zhang and co-workers [30] failed due to the low expression of E- and N-cadherin on the surface of RCC cells (data not shown).

Most of the RCC cell lines showed elevated mesenchymal markers and reduced epithelial markers after 96 h in the absence of exogenous TGF-β1 (Figure 6B, Supplementary Figure 3). The 786-O cells showed heterogeneous results: Although the E-cadherin levels were decreasing after re-culturing of the cells indicating a stronger mesenchymal transition, the expression of the mesenchymal marker *MMP2* is fully repressed. In contrast, decreased mRNA levels of *CDH1* and increased mRNA levels of *MMP2* were found in all other cell lines in comparison to unstimulated RCC cells even after 96 h without exogenous stimulus. However, the extent of the epithelial repression and enhanced mesenchymal gene expression was lower in most cases when compared to the expression levels after 96 h of TGF-β1 stimulation.

For the one representative ccRCC cell line MZ2733RC and the pRCC cell line MZ2858RC, the MMP2 mRNA levels directly correlate with endogenous *TGFB1* mRNA levels. If the *TGFB1* level dropped after re-culturing in comparison to the level directly after the 96 h stimulation (Figure 6B, dark grey bars), the *MMP2* mRNA level decreased likewise. When the *TGFB1* levels increased even in the absence of the exogenous stimulus, the *MMP2* simultaneously increased as demonstrated for MZ2733RC. In conclusion, most RCC cell lines remain in the mesenchymal status after removal of the external stimulus due to endogenous TGF-β1 production.

### Blockade of the TGF-β/Smad signaling pathway in RCCs by the TGFBR1 inhibitor SB431542

**Figure 7:**
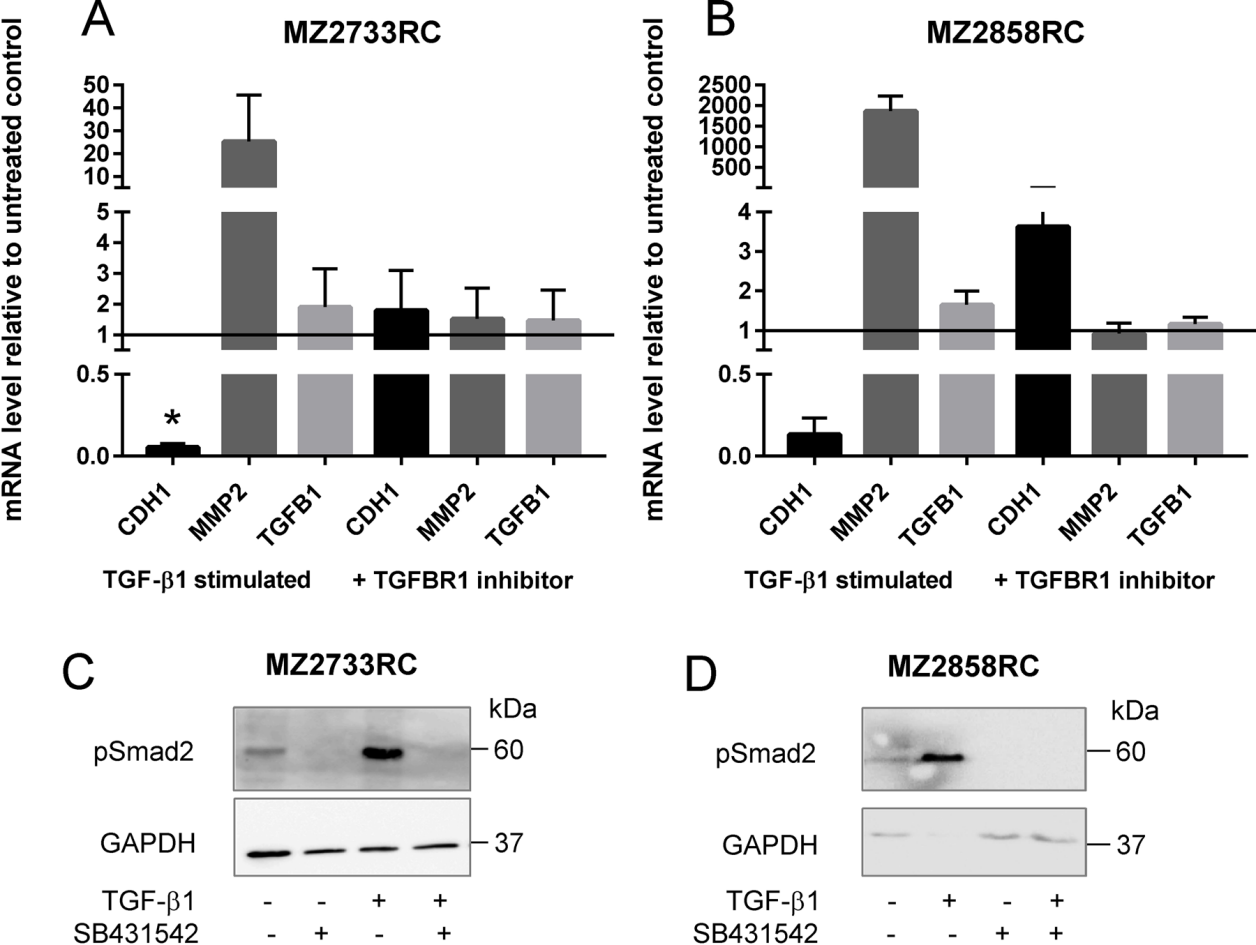
Inhibition of the TGF-β/Smad signaling pathway in RCCs (**A–B**) Bar graphs show mean values of 3 independent experiments (*n* = 3). *CDH1* represents the epithelial marker E-cadherin (black bars). Dark grey bars indicate the mRNA levels of the mesenchymal marker *MMP2*; the light grey bars show *TGFB1* mRNA levels. All values are displayed as values relative to the untreated control after 96 h of TGF-β1 treatment and simultaneous treatment with TGF-β1 and inhibitor, respectively. (**C–D**) Representative Western blot analysis of phosphorylated Smad2 showed the presence of the protein after TGF-β1 treatment. No signal was detected in the presence of the TGFBR1 inhibitor SB431542. GAPDH detection serves as loading control. (^*^p ≤ 0.05).

Simultaneous addition of TGF-β1 and the TGFBR1 inhibitor SB431542 efficiently blocks the TGF-β/Smad signaling pathway. Thus, the RCC cells retain their epithelial phenotype characterized by neither a decrease of *CDH1* nor an increase of *MMP2* in the presence of TGF-β1 and the inhibitor (Figure 7A, 7B). Additionally, no phosphorylated Smad2 was detected upon addition of TGF-β1 and the TGFBR1 inhibitor (Figure 7C, 7D).

### Lack of MET induction by the TGFBR1 inhibitor SB431542

**Figure 8:**
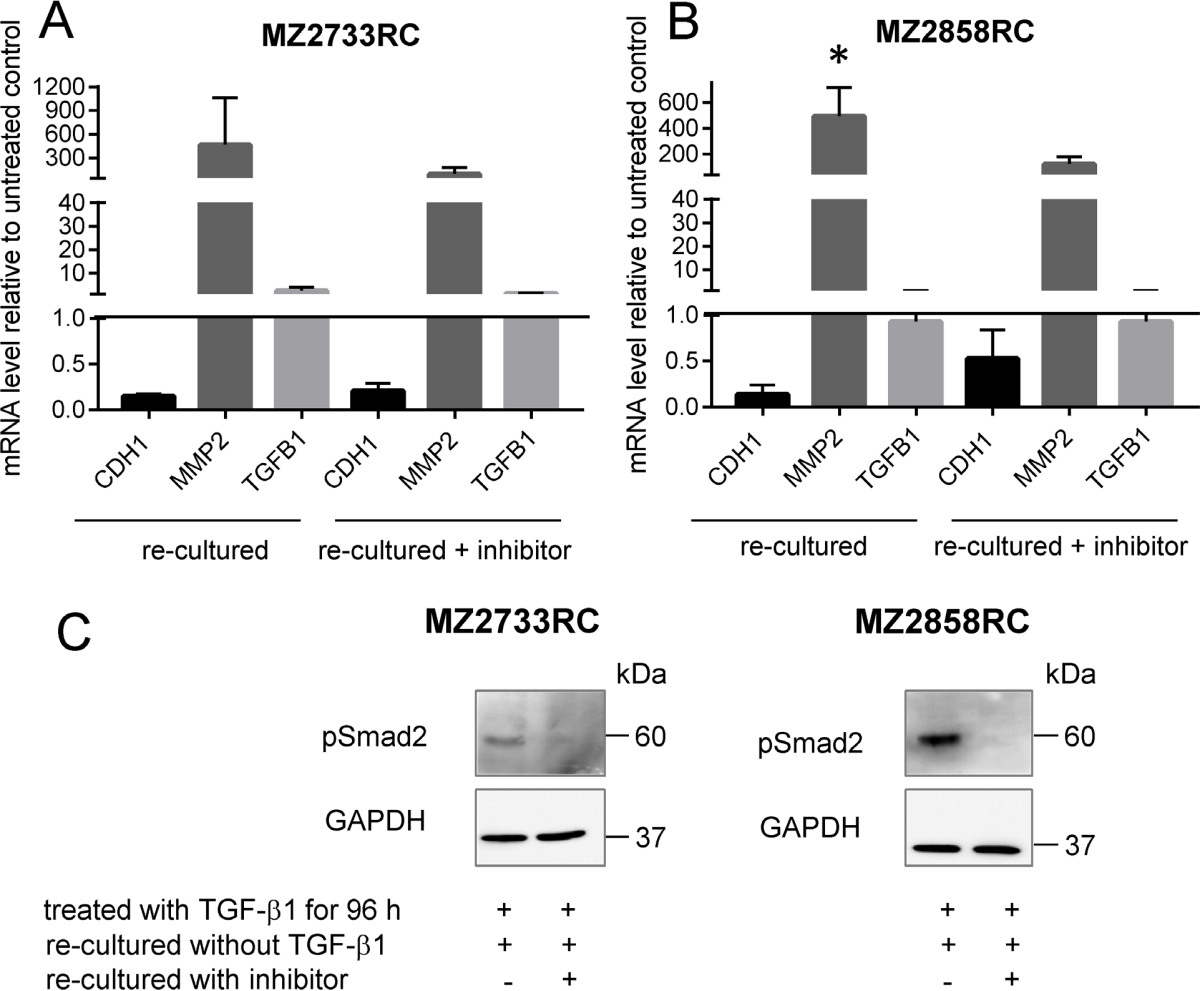
Re-culture and inhibition experiment with RCCs (**A–B**) Bar graph shows representative data of two independent reproducible experiments. Black bars display the mRNA levels of the epithelial marker *CDH1*. Dark grey bars indicate the mRNA levels of the mesenchymal marker *MMP2*; the light grey bars show *TGFB1* mRNA levels. All values are indicated as mean values relative to the untreated control after 96 h of TGF-β1 treatment and subsequent treatment with TGFBR1 inhibitor, respectively. (**C**) Representative Western blot analysis of phosphorylated Smad2 during re-cultured experiment. Signal is present in the absence of the inhibitor; no signal was detected in the presence of the TGFBR1 inhibitor SB431542. GAPDH detection serves as loading control. (^*^p ≤ 0.05).

After transition of the RCC cells to the mesenchymal cell type, the reversibility of this process was determined by blocking the TGFBR1 with the inhibitor SB431542. Therefore, the cells were first treated with TGF-β1 and subsequently re-cultured with medium containing the inhibitor SB431542. Interestingly, once transitioned to the mesenchymal cell type, the cells do not fully revert to an epithelial cell type even though the TGF-β/Smad signaling pathway was subsequently blocked with the TGFBR1 inhibitor (Figure 8). As shown by qPCR, the mRNA level of the epithelial marker increases and the one of the mesenchymal decreases in the presence of the inhibitor in comparison to the re-culturing without inhibitor for both cell lines tested (Figure 8A, 8B). No difference in endogenous *TGFB1* mRNA levels was observed for re-culturing with or without inhibitor. Western blot analysis showed a signal for pSmad2 when cells were re-cultured without inhibitor for both cell lines. No pSmad2 was detected in the presence of the inhibitor during the re-culture experiment (Figure 8C).

**Figure 9:**
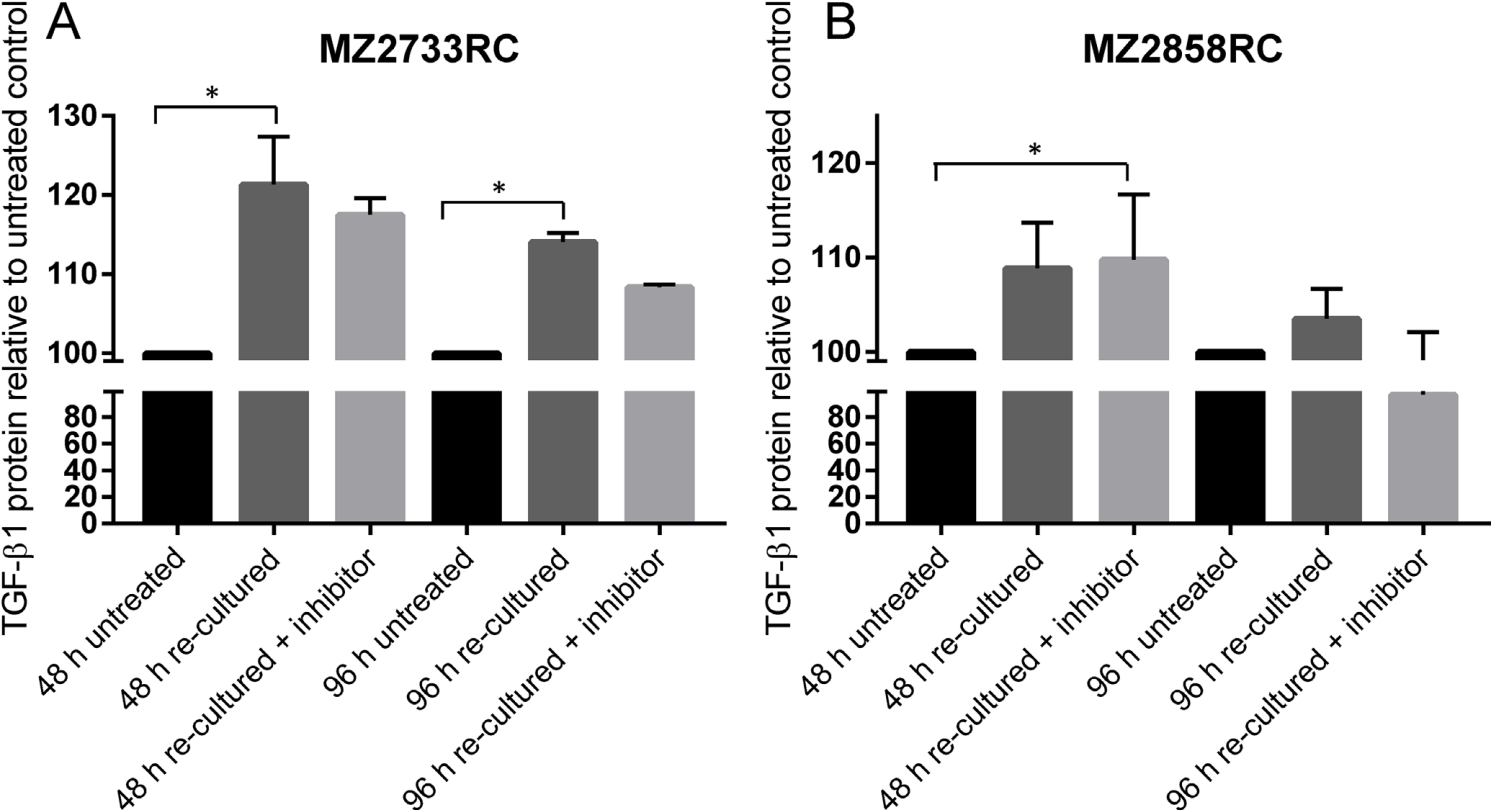
Determination of secreted TGF-β1 from stimulated RCC cells (**A–B**) Bar graphs show mean values of 3 measurements (*n* = 3). Secreted TGF-β1 is displayed as protein level relative to the untreated control. Both cell lines show elevated TGF-β1 protein levels during the re-culturing period of 96 h. Re-culturing in the presence of the TGFBR1 inhibitor decreases the secreted TGF-β1 protein in the case of MZ2733RC. For MZ2858RC, no secreted TGF-β1 protein was detectable any more after 96 h in the presence of the inhibitor. (^*^p ≤ 0.05).

To check not only for *TGFB1* mRNA but for secreted TGF-β1 protein, supernatants were analyzed using enzyme-linked immunosorbent assay (ELISA) with an antibody against TGF-β1. The medium was changed after 96 h of TGF-β1 stimulation and cells were re-cultured in the absence and presence of the TGFBR1 inhibitor SB431542. In general, elevated TGF-β1 protein levels were detected for cells that were stimulated with external TGF-β1 in comparison to untreated cells (Figure 9A, 9B). After the first 48 h of re-culturing, the TGF-β1 protein levels were higher than after a second period of 48 h re-culturing (96 h in total) when medium was changed after the first 48 h. In the presence of the inhibitor, the TGF-β1 protein level was lower in comparison to the experiment in the absence of the inhibitor. For MZ2858RC, the TGF-β1 protein level reverted to the level of untreated cells after 96 h re-culturing in the presence of the inhibitor indicating that no TGF-β1 protein was secreted anymore into the supernatant by the pRCC cells (Figure 9B).

## DISCUSSION

Epithelial-to-mesenchymal transition is a crucial process that leads to cancer development and progression through metastasis formation resulting in a worse prognosis and overall survival of patients with RCC (reviewed in [43]). In RCC, EMT can be induced by a variety of factors, such as TNF-α [20], oxidative stress [44], loss of VHL [45] or FOXO3A [27], and deregulation of miRNAs [46, 47]. However, a detailed study of the effect of one major EMT-inducer – the transforming growth factor beta (TGF-β) - on renal cell carcinoma remained elusive up to now.

Here, the impact of TGF-β on two different RCC subtypes was analyzed on cell line level. Five different ccRCC cell lines and one pRCC cell line were found to transition to a mesenchymal cell type upon treatment with recombinant TGF-β1. Since TGF-β1 mainly exerts its cellular effects by the Smad-signaling pathway [48, 49], the functionality of this pathway was analyzed in the different RCC cell lines. The signaling cascade is induced through binding of TGF-β1 to its cognate receptor subunit II (TGFBR2) which is constitutively active and activates the kinase domain of the receptor subunit I (TGFBR1) [50]. Treatment of different RCC cell lines with recombinant TGF-β1 showed a down-regulation of *TGFBR2* while *TGFBR1* was upregulated (Figure 2A) with exception of the ccRCC cell line MZ2733RC, which showed a down-regulation for both receptor subunits. It was found that loss of one *TGFBR2* allele is associated with tumor progression and metastasis [51]. Furthermore, a tumor-suppressive role for an intact TGFBR2 and signaling pathway was shown in mice [52, 53]. Since low levels of TGFBR2 are associated with poor prognosis [54], our findings of *TGFBR2* down-regulation are in line with previous studies. These data were further underlined by the survival of RCC patients according to Kaplan–Meier curves [55], which showed a significantly lower overall survival in ccRCC patients with low levels of *TGFBR2* in comparison to the ones with high expression of *TGFBR2* (Supplementary Figure 4).

The levels of TGFBR2 were shown to regulate the downstream signaling pathway: high expression induces the Smad-dependent signaling pathway while a low expression triggers signaling via the non-Smad-dependent MAP/ERK pathway in colon cancer [56]. Our findings revealed the exact opposite. Although the RCC cell lines showed a low expression of the *TGFBR2* after treatment with recombinant TGF-β1, we detected the induction of the Smad-dependent pathway by high levels of the phosphorylated Smad2 protein (pSmad2) and the downstream target *MMP2* in comparison to untreated RCC cells (Figure 2B, 2C). However, the impact of TGF-β1 on other signaling pathways was not investigated in this study and requires more detailed research.

Elevated levels of pSmad2 and *MMP2* indicate that TGF-β1 exerts its biological function through the TGF-β1/Smad-signaling pathway in both ccRCC and pRCC. The analysis of morphology, migration and EMT marker expression revealed that all cell lines tested undergo the EMT process (Figures 1, 3, and 4). Although the transition occurred to different extents, all cell lines followed the hallmarks of EMT: repression of E-cadherin through increasing expression of transcriptional repressors of E-cadherin such as Snail, Slug, and ZEB1. Additionally, the mRNA levels of typical mesenchymal markers such as vimentin and N-cadherin were elevated for most of the cell lines tested. Thereby the extent of fold change in epithelial marker repression and mesenchymal marker expression is comparable to the findings with TNF-α treatment [20] or oxidative stress [44] in RCCs as well as TGF-β1 treatment of other cell lines such as non-tumorigenic mammary MCF-10 [30], bronchial epithelial cell lines [57], and the alveolar epithelial cell line A549 [58]. Comparing the morphology change and the extent of EMT marker repression/expression, two cell lines responded best to the TGF-β1 treatment: the ccRCC cell line MZ2733RC and the pRCC cell line MZ2858RC, which were used for further experiments. Up to now, differences and similarities of the EMT process in different RCC subtypes are still not well characterized. Morra and co-workers showed that levels of the matrix N-glycoprotein periostin that can promote EMT are higher in ccRCC than in the papillary type which correlated with tumor grading and staging as well with poor overall survival [59]. Both ccRCC and pRCC are capable of differentiation into the sarcomatoid type of RCC with highly malignant and aggressive properties and worse prognosis [60]. Here, we show that TGF-β1 efficiently induces EMT in both RCC subtypes. All analyzed parameters – morphology change, wound healing properties, EMT marker pattern, and induction of the Smad-signaling pathway – were comparable for the clear cell and the papillary cell type. However, a correlation of high *TGFB1* levels and worse overall survival was only determined for the clear cell but not for the papillary type of RCC (Supplementary Figure 5). This correlation is underlined by the clinical data published for ccRCC by Sitaram and co-workers [19]. In summary, our findings are in line with previous data showing that higher *TGFB1* levels lead to poor prognosis. The underlying mechanism is the induction of EMT by TGF-β1 protein through the TGF-β/Smad-signaling pathway in ccRCC and pRCC cells, respectively.

Besides similarities in EMT induction, we detected differences in surface expression of immune modulatory molecules for the representative ccRCC cell line MZ2733RC and the pRCC cell line MZ2858RC. If this is a subtype or simply a cell line specific expression can only be speculated. Therefore, more cell lines of different subtypes would need to be investigated. Furthermore, heterogeneities of surface molecule expression within tumors were found for PD-L1 [61] indicating that cell lines generated from the same tumor but different areas might already differ in surface molecule expression. However, we found that HLA-ABC, a component of the antigen presenting machinery, was mainly not or down-regulated upon TGF-β1 stimulation (Figure 5). This is in accordance with the general understanding of immune evasion of tumors during malignant transformation [62] and is underlined by the down-regulation of different APM components on mRNA level upon TGF-β1 treatment (Supplementary Figure 6). In contrast to MZ2733RC, which showed an upregulation of the co-stimulatory molecules B7-H2 and B7-H3, the pRCC cell line down-regulated their expression upon TGF-β1 stimulation. While B7-H2 is a co-stimulatory molecule promoting T cell activation [63], a dual role in inhibition and activation of T cells was described for B7-H3, respectively [64–66]. Both cell lines showed a down-regulation of ICAM-1 (intercellular adhesion molecule 1) after TGF-β1 stimulation. ICAM-1 was shown to be a ligand for LFA-1 (β_2_ integrin lymphocyte function-associated antigen 1), a receptor expressed on leukocytes [67, 68] facilitating leukocyte endothelial transmigration. The down-regulation of ICAM-1 after TGF-β1 stimulation underlines the tumor’s evasion from the immune system by reducing immune cell infiltration into the tumor microenvironment and tumor cell elimination by the tumor infiltrating lymphocytes (TILs).

**Figure 10:**
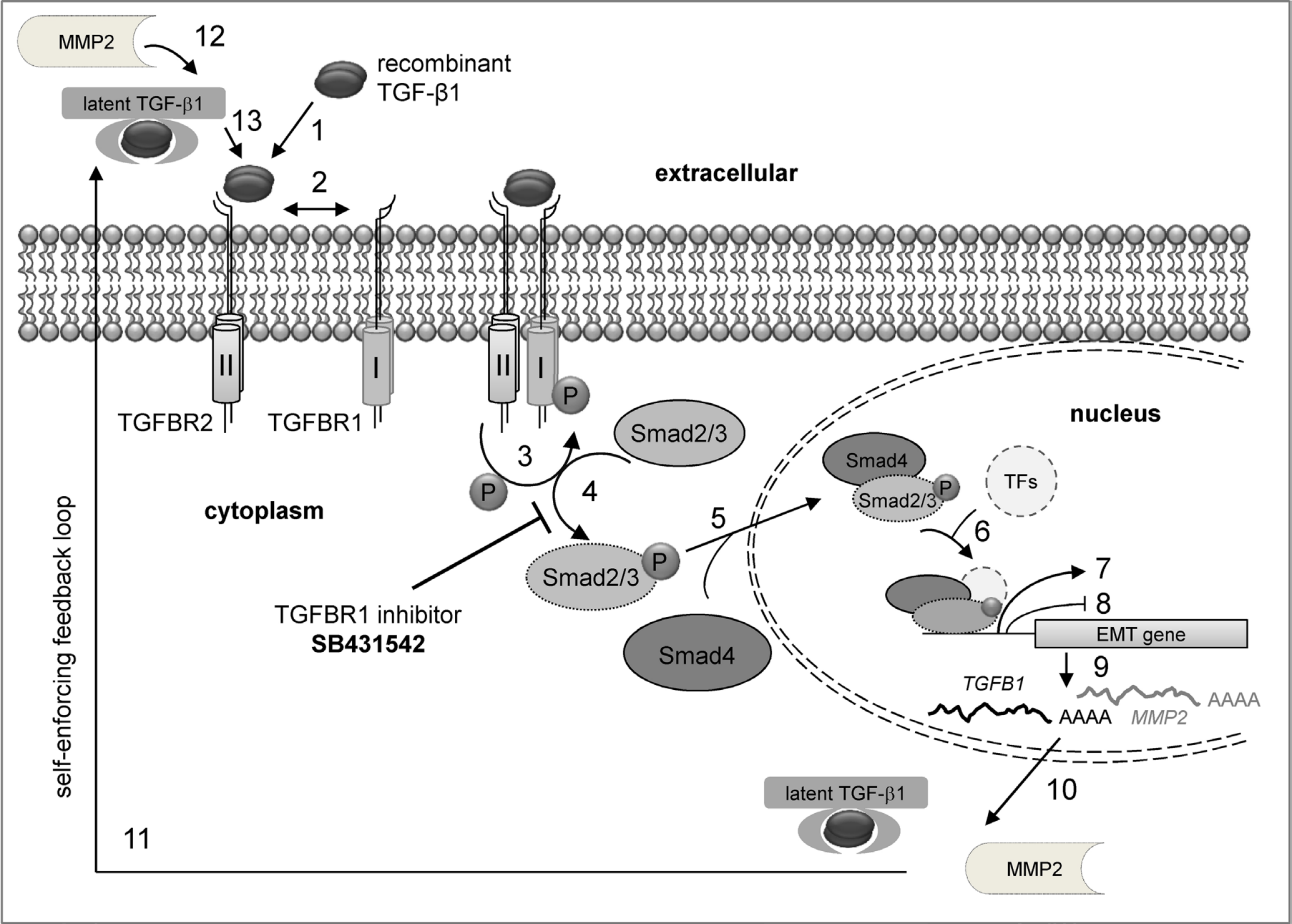
Model for the TGF-β/Smad signaling feedback loop after external TGF-β stimulation (**1**) Binding of recombinant TGF-β1 to TGFBR2. (**2**) Receptor activation and heterotetramer formation by TGFBR1 and TGFBR2 dimers. (**3**) Phosphorylation of TGFBR1 by TGFBR2. (**4**) Stimulation of the Smad signaling pathway by Smad2/3 phosphorylation via TGFBR1. This step is efficiently blocked by the inhibitor SB431542 resulting in loss of Smad2/3 phosphorylation. (**5**) Association of Smad4 with p-Smad2/3 and transport to the nucleus. (**6**) Binding of the p-Smad2/3-Smad4 complex to transcription factors (TFs). (**7**) Enhanced mesenchymal gene expression and (**8**) repression of epithelial gene expression. (**9**) Messenger RNA production of mesenchymal genes. (**10**) Transport of mRNA and translation of mesenchymal proteins in the cytoplasm. (**11**) Secretion of latent TGF-β and matrix metalloproteases (e.g. MMP2). (**12**) Cleavage of latent TGF-β e.g. by MMP2. (**13**) Binding of active TGF-β to its cognate receptor.

Although all RCC cell lines investigated in this study transitioned to the mesenchymal cell type, the EMT transition status and reversibility of the EMT process were of great interest. It was shown that distinct factors such as WT1 (Wilms tumor protein 1) can induce hybrid EMT states (EMHT, upregulated Snail, maintained E-cadherin expression [69]). In this context the transition status of five ccRCC cell lines and one pRCC cell line were investigated using re-culture experiments. Shifting the cells to medium lacking the external stimulus after initial TGF-β1 treatment did not restore the epithelial cell type (Figure 6, Supplementary Figure 3). This can be explained by elevated endogenous *TGFB1* mRNA levels even after shifting the cells back to medium without the external TGF-β1 stimulus. Obviously, the treatment with recombinant TGF-β1 protein leads to a self-enforcing feedback loop (Figure 10). Once stimulated with TGF-β1, the RCC cells start to produce and secrete their own TGF-β1 as shown by elevated *TGFB1* mRNA and protein levels (Figures 6 and 9). The secreted latent form can be cleaved by e.g. the matrix metalloproteases such as MMP2 (Figure 10) which was highly upregulated after TGF-β1 treatment (Figure 2). The intracellular signaling cascade was efficiently blocked upon addition of the TGFBR1 inhibitor SB431542 since no phosphorylated Smad2 was detected in the presence of the inhibitor (Figure 7). Shifting the RCC cells to medium without TGF-β1 but containing the inhibitor showed an efficient block of the TGF-β/Smad-signaling pathway since no phosphorylated Smad2 was detectable (Figure 8C). Furthermore, no elevated TGF-β1 protein levels were observed after 96 h of re-culturing in the presence of the inhibitor for the MZ2858RC cell line (Figure 9B). For MZ2733RC slightly higher TGF-β1 protein levels relative to the untreated control were detected after 96 h inhibitor treatment. Furthermore, the cells did not fully revert to the epithelial cell type as shown for the EMT markers *CDH1* and *MMP2* on mRNA level: *CDH1* was still repressed and *MMP2* higher expressed than the untreated controls (Figure 8A and 8B). Although the Smad-dependent pathway was fully blocked in the presence of the inhibitor as shown by Western blot, the activation of transcription factors can also be accomplished by Smad-independent pathways. Furthermore, EMT can be induced by a variety of other factors such as fibroblast growth factor (FGF), bone morphogenetic protein (BMP), platelet-derived growth factor (PDGF), epidermal growth factor (EGF), Sonic Hedgehog (Shh), Notch, integrin, and Wnt signaling via several different signaling cascades [70–76]. Since we did not analyze the presence of other EMT inducing factors in the supernatants of the RCC cell lines, we cannot exclude that the mesenchymal cell type is maintained by a different mechanism than the TGF-β/Smad-signaling pathway in the presence of the inhibitor.

In summary, we developed a TGF-β1 inducible model system to study the process of EMT in RCC. Uncovering the basics of EMT in RCC is essential to gain insights into the mechanism of metastasis and tumor evasion from the immune system. Therefore, our model system provides a solid basis for more detailed research of EMT in RCC and brings TGF-β1 into focus as putative target for anti-tumor therapies.

## MATERIALS AND METHODS

### Cell lines and culture conditions

The 786-O cell line was kindly provided by Prof. Wiesner (Erlangen). Caki-1 (ATCC^®^HTB-46™) and Caki-2 (ATCC^®^HTB-47™) were purchased from the American Type Culture Collection (ATCC, Manassas, VA, USA). MZ1851RC, MZ2733RC, and MZ2858RC were generated from primary tumors of the clear cell or papillary subtype as previously described [77, 78]. All RCC cell lines were cultured in Dulbecco’s Modified Eagle Medium (DMEM, #11965092, Thermo Fisher, Waltham, MA, USA) supplemented with 10% (v/v) fetal calf serum, 1% (v/v) penicillin-streptomycin, 1% (v/v) minimal-essential medium non-essential amino acids (MEM NEAA), and 1% (v/v) sodium pyruvate.

### Stimulation, re-culture and inhibition experiments

RCC cells were seeded in 100 mm cell culture dishes 24 h prior stimulation. For TGF-β1 stimulation, the cells were grown for 96 h in the presence of 10 ng/mL TGF-β1 (Biolegend, San Diego, CA, USA, Cat. No. 580704). For TGFBR1 inhibition, the cells were treated with 10 ng/mL SB 431542 (Sigma, St. Louis, MO, USA, CAS no. 301836-41-9) for 96 h. To refresh the stimulus or inhibitor, the medium was changed once after 48 h. For re-culture experiments, the cells were shifted to medium without TGF-β1 or DMEM supplemented with 10 ng/mL SB 431542 for 96 h after 96 h TGF-β1 treatment. Analogous, the medium was refreshed after 48 h. Untreated cells grown for the same period of time served as unstimulated controls.

### Cell viability, proliferation and apoptosis assay

The cell viability was assayed prior and after addition of 10 ng/mL TGF-β1 over a period of 96 h using a Muse cell analyzer (Merck Millipore, Burlington, MA, USA). Viable and non-viable cells were differentially stained based on their permeability to the DNA-binding dyes in the reagent. Cell proliferation was determined by measuring the conversion of tetrazolium salt (XTT) to formazan using the Cell Proliferation kit II (Roche Applied Science, Penzberg) as recently described [79]. To determine the apoptosis rate, fluorophore-coupled annexin V detecting apoptotic cells by binding to their cell membrane was used, whereas 7-aminoactinomycin D (7AAD) binding to the DNA served as marker for dead cell discrimination. This combination allows the differentiation among early apoptotic cells (annexin V positive, 7AAD negative), necrotic cells (annexin V positive, 7AAD positive), and viable cells (annexin V negative, 7AAD negative).

### Wound healing assay

Wound healing assays were performed according to previous descriptions [80]. In brief, the ccRCC cell type (MZ1851RC) and the pRCC cell type (MZ2858RC) were grown in 6-well plates +/- 10 ng/mL TGF-β1 for 96 h to 100% confluence. A 200 μL tip was used to set a scratch in the monolayer surface. After setting the scratch, the medium was changed to low serum level medium (DMEM + supplements + 0.5% FCS) to reduce proliferation. Images were taken at indicated time points using a BD Pathway 855 system. Area of scratch was determined using ImageJ software.

### RNA isolation, cDNA synthesis and quantitative PCR

For the isolation of total RNA from different RCC cell lines the NucleoSpin^®^ RNA kit from Macherey-Nagel (Dueren) was used according to the manufacturer’s protocol. One μg of the total RNA was used for cDNA synthesis with random hexamer primers using the Thermo Scientific RevertAid First Strand cDNA Synthesis kit according to the manufacturer’s instructions. The quantification of gene expression was determined by quantitative PCR using SYBR Green qPCR Master Mix (Bimake, Houston, TX, USA) in a BioRad CFX Connect cycler. Real time quantitative PCR amplifications were performed in a final volume of 10 µl with an initial denaturation and polymerase activation step of 7 min at 95°C followed by 40 cycles with denaturation at 95°C for 10 s and annealing/elongation in one step at 60°C for 30 s. All reactions were run as triplicates of biological replicates. Differential expression was analyzed using the ΔΔCt method. The mRNA expression of genes of interest was normalized to the mean of glyceraldehyde 3-phosphate dehydrogenase (*GAPDH*) and β-actin (*ACTB*) mRNA expression and described as mRNA levels relative to the untreated controls. The mRNA-specific qPCR primers are listed in the Supplementary Table 1.

### Protein extraction and Western blot analyses

From the same cells previously used for RNA isolation, proteins were extracted and the concentration was determined with the Pierce BCA protein assay kit (Thermo Fisher). 50 μg protein per lane were separated on 10–12% denaturing polyacrylamide gels, transferred onto nitro cellulose membranes (GE Healthcare, Chicago, IL, USA) and stained with Ponceau S. Membranes were incubated with primary antibodies specific for Smad2 (#3103S, Cell Signaling, Danvers, MA, USA), phosphorylated Smad2 (#3108S, Cell Signaling), and GAPDH (2118S, Cell Signaling) (1:1000 to 1:5000 diluted TBS-T, 5% (w/v) bovine serum albumin (BSA)) overnight at 4°C. For detection, a secondary antibody conjugated with horseradish peroxidase (HRP) and the Lumi-Light substrate (Roche Applied Science) were applied. Proteins were analyzed as triplicates of biological replicates. Protein band intensities were determined using ImageJ software. Protein levels were normalized to the housekeeper GAPDH and displayed as values relative to the untreated control.

### Flow cytometry

The monoclonal antibodies used for flow cytometry against various surface molecules are listed in Supplementary Table 2 and were fluorescein isothiocyanate-labeled (FITC), phycoerythrin-labeled (PE), and allophycocyanine-labeled (APC), respectively. Respective labeled isotype mouse immunoglobulins IgG1 and IgG2a served as isotype controls. Cells were analyzed for surface molecule expression after 96 h growth in the presence and absence of 10 ng/mL TGF-β1. Cells were harvested with trypsin, washed with 1x PBS and 40.000 cells were incubated with the respective antibody for 45 min on ice. Stained cells were washed twice with 1x PBS and analyzed using a BD Fortessa flow cytometer. The results are expressed as mean specific fluorescence intensity obtained from at least three independent experiments.

### Enzyme-linked immunosorbent assay (ELISA)

Supernatants from re-culturing experiments were collected, spun down at 10.000 x g to remove cells and cell debris, upper soluble part was flash frozen in liquid nitrogen and stored at –80°C. Samples were thawed and latent TGF-β1 was activated under acidic conditions according to the manufacturer’s recommendations. ELISA procedure was carried out following the manufacturer’s instructions (Quantikine ELISA, R&D Systems, Minneapolis, MN, USA). After enzyme reaction, absorbance was measured at 450 nm and 540/570 nm for wavelength correction using a TECAN microplate reader (Tecan, Switzerland).

### Bioinformatical and statistical analyses

Statistical analysis was performed using Graph Pad Prism v.7.00. The comparisons within the experiments were conducted using the nonparametric (non-Gaussian) distribution assumption given the small size of the sample, repeated measures version of ANOVA, and Friedman test. As post hoc analysis Dunn’s test was included, in order to distinguish the significant differences between the group ranks attributed by Friedman test. The values used for these tests, were the raw Δct values, to determine the differences between the treated samples and their respective control groups. Individual *t*-tests were carried out on IBM SPSS 25.0. Equal variances were taken into consideration, when Lavene’s test for equality of variances allowed it.

Kaplan–Meier curves to display overall survival in correlation to different gene expressions were performed using Xena Browser (https://xenabrowser.net/). Datasets were obtained from “The Cancer Genome Atlas” (https://portal.gdc.cancer.gov) with 604 patients for TCGA-KIRC (ccRCC) and 320 patients for TCGA-KIRP (pRCC).

## SUPPLEMENTARY MATERIALS FIGURES AND TABLES



## Abbreviations

APM: antigen presenting and processing machinery
B7-H: homolog of B7 co-stimulatory ligand
ccRCC: clear cell renal cell carcinoma
CDH1: E-cadherin
CDH2: N-cadherin
CLDN1: claudin 1
chRCC: chromophobe renal cell carcinoma
ECM: extracellular matrix
ELISA: enzyme-linked immunosorbent assay
EMT: epithelial-to-mesenchymal transition
HLA: human leucocyte antigen
ICAM: 1 intercellular adhesion molecule 1
LFA-1: β_2_ integrin lymphocyte function-associated antigen 1
mAb: monoclonal antibody
MHC: major histocompatibility complex
MMP: matrix metalloprotease
PD-L1: programmed death protein ligand 1, alias B7-H1
pRCC: papillary renal cell carcinoma
SNAI1: Snail transcription factor
SNAI2: Slug transcription factor
TF: transcription factor
TGF-β: transforming growth factor beta
TGFBR: transforming growth factor beta receptor
TIM3: T-cell immunoglobulin and mucin-domain containing-3
TME: tumor microenvironment
TNF-α: tumor necrosis factor alpha
VIM: vimentin
VHL: von Hippel-Lindau gene
ZEB1: Zinc finger E-box-binding homeobox 1 transcription factor
