# Supplementary material for: TGF-β inducible epithelial-to-mesenchymal transition in renal cell carcinoma
Source: Oncotarget. 2019 Feb 19;10(15):1507–24. doi: 10.18632/oncotarget.26682 (PMC6407676; doi:10.18632/oncotarget.26682)
Supplement: Supplementary file 1 [file oncotarget-10-1507-s001.pdf]

# TGF-β inducible epithelial-to-mesenchymal transition in renal cell carcinoma

## SUPPLEMENTARY MATERIALS

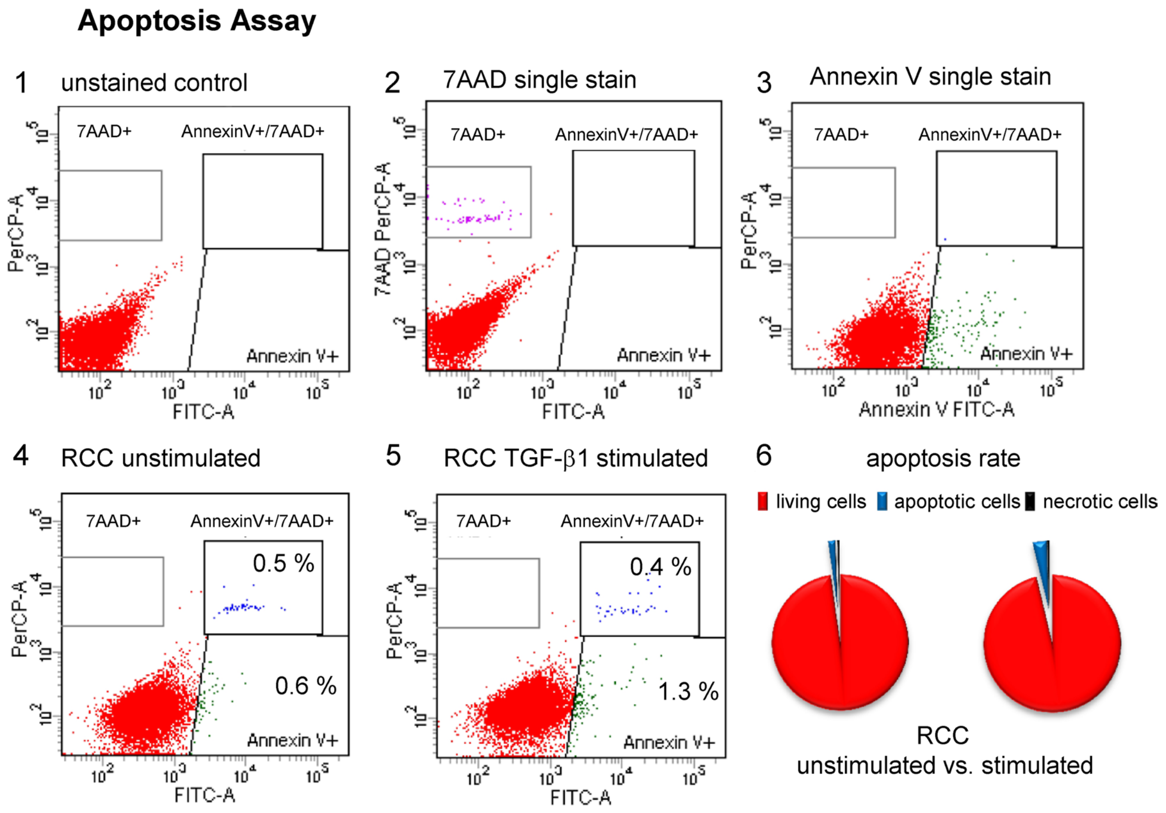


**Supplementary Figure 1: Influence of TGF-β1 treatment on apoptosis.** RCC cells were stained with 7AAD and annexin V and the frequency of stained cells as a measure of apoptosis or necrosis was determined over time. Diagrams 1–5 show the fluorescence intensity of annexin V-FITC on the x-axis and 7AAD-PerCP on the y-axis. Unstained control (1) and single stains (2, 3) were required for gating the cells and analyze TGF-β1-stimulated vs. unstimulated RCCs. Pie diagrams (6) show apoptosis rates of one representative experiment with 96 h TGF-β1- stimulated and unstimulated RCC cells.

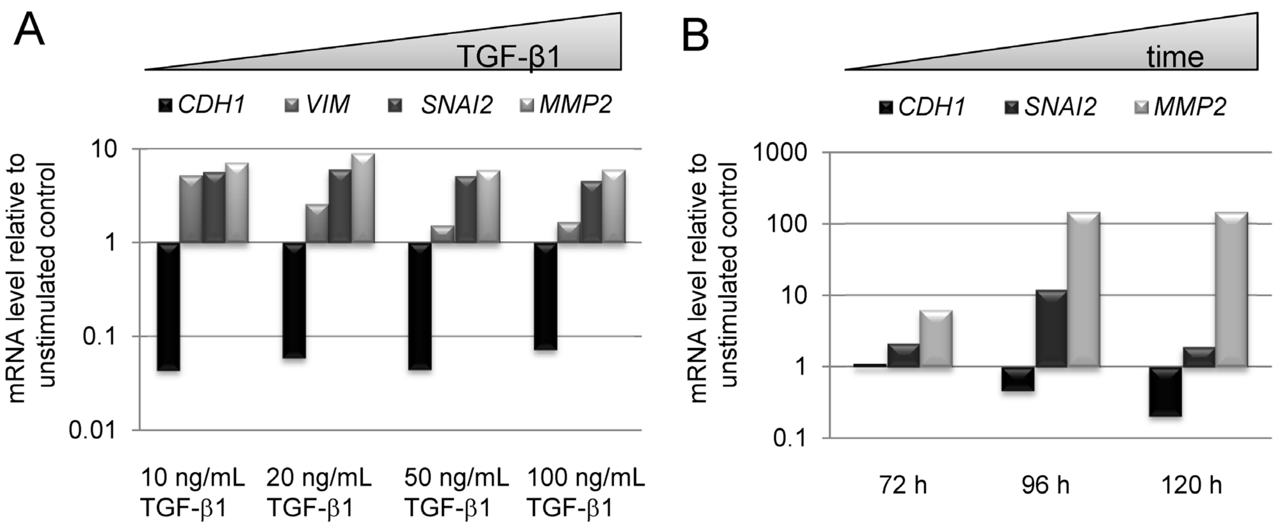


**Supplementary Figure 2: Effect of increasing amounts and interval of TGF-β1 treatment on renal cell lines.** *CDH1* repression and upregulation of mesenchymal markers were investigated via qPCR. Data from one representative experiment are shown, respectively. (**A**) EMT markers do not increase or decrease more with increasing amounts of TGF-β1 in MZ2733NN. 10 ng/mL appeared to be a suitable concentration of TGF-β1 to trigger EMT in renal cell lines. (**B**) MZ2858RC cells were treated for 72 h – 120 h with 10 ng/mL TGF-β1. Although the repression of *CDH1* and the expression of the mesenchymal markers seem to increase from 72 h to 96 h, no remarkable increase from 96 h to 120 h stimulation time was detected. Therefore, for RCC stimulation experiments, a treatment with 10 ng/mL TGF-β1 for 96 h was determined to be the method of choice.

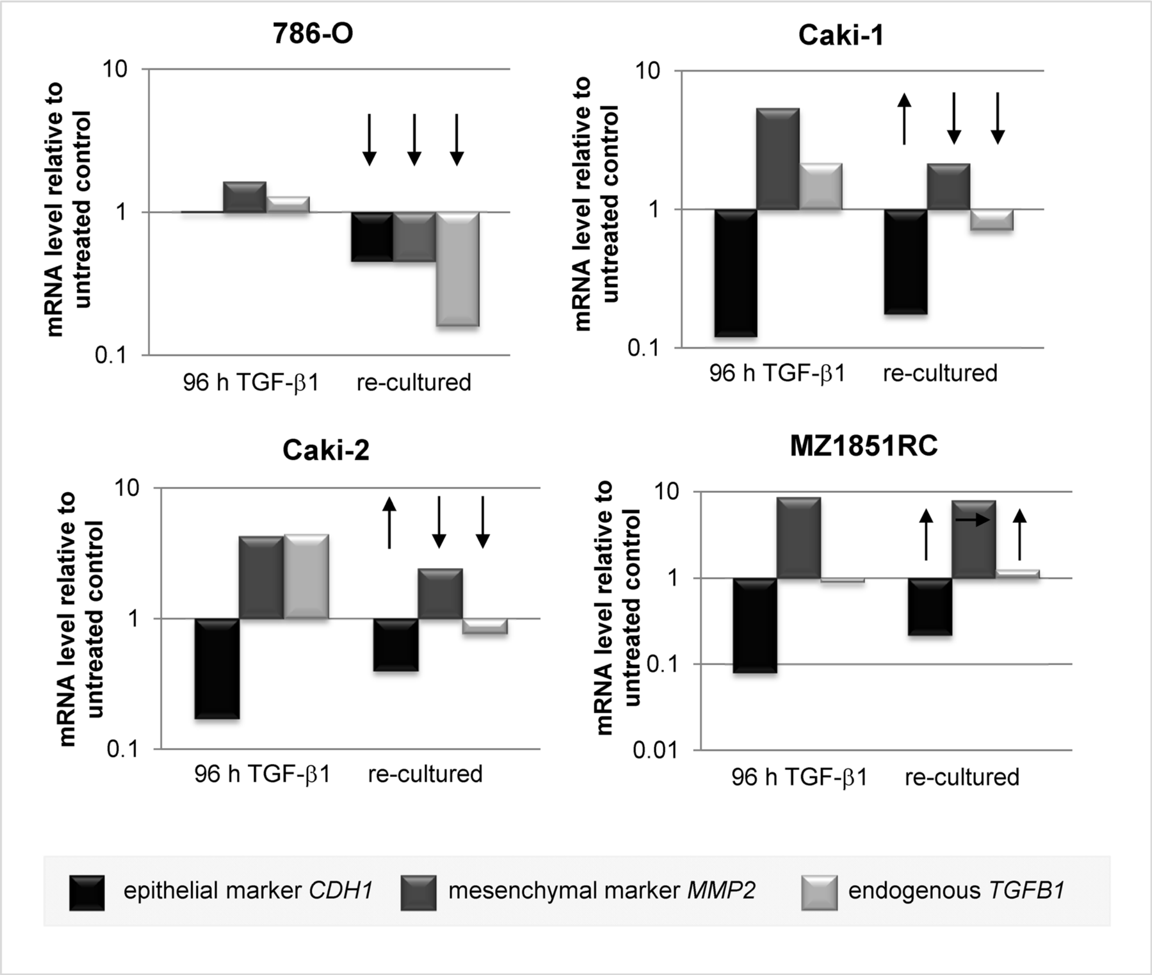


**Supplementary Figure 3: Reversibility of the mesenchymal transition of RCC cells analyzed by qPCR.** Bar graphs show data of one representative re-culturing experiment. RCC cell lines were tested for the reversibility of the mesenchymal transition after 96 h by re-culturing in medium without TGF-β1. Black bars display the mRNA levels of the epithelial marker *CDH1* relative to the untreated control after 96 h of TGF-β1 treatment and additional 96 h re-culturing, respectively. Dark grey bars indicate the mRNA levels of the mesenchymal marker *MMP2*; the light grey bars show *TGFB1* mRNA levels. Arrows label the increase (↑), decrease (↓) or no change (→) of mRNA levels of the respective genes after re-culturing.

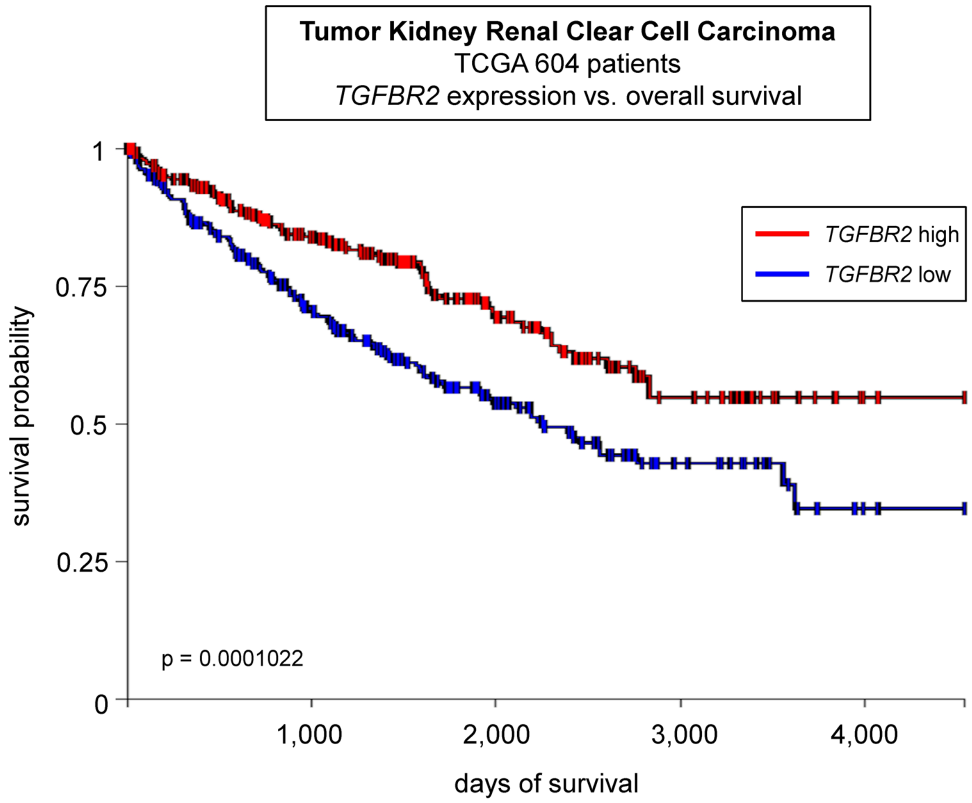


**Supplementary Figure 4: Kaplan-Meier curve for overall survival of ccRCC patients with different *TGFBR2* expression levels.** Patients with high *TGFBR2* expression ($n = 301$, red) have a significantly better overall survival ($p$-value = 0.0001022) than patients with low *TGFBR2* expression levels ($n = 303$, blue).

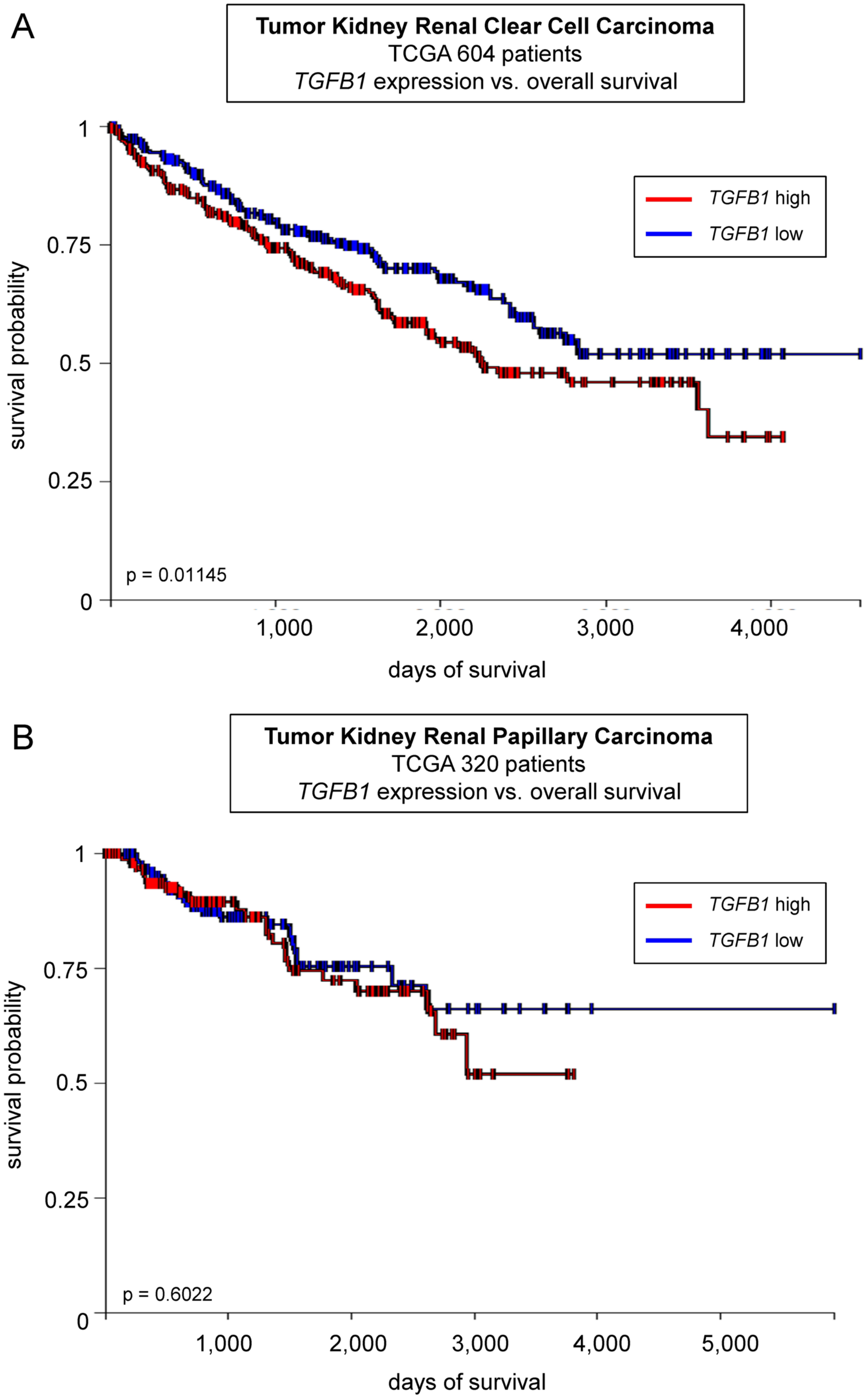


**Supplementary Figure 5: Kaplan-Meier curve for overall survival of ccRCC and pRCC patients with different *TGFB1* expression levels.** (**A**) Clear cell RCC patients with low *TGFB1* expression ($n$ = 302, blue) have a significantly better overall survival ($p$-value = 0.01145) than patients with high *TGFB1* expression levels ($n$ = 302, red). (**B**) No significant difference in overall survival was determined for pRCC patients with different *TGFB1* expression levels.

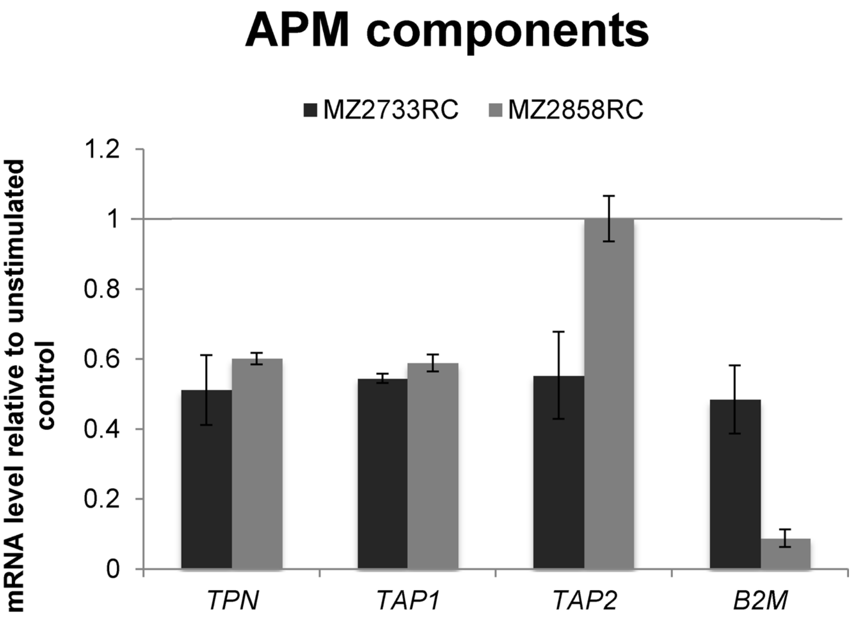


**Supplementary Figure 6: The effect of the TGF-β1 treatment on the mRNA levels of components of the antigen presenting and processing machinery.** Bar graphs show qPCR data of at least 3 independent stimulation experiments ($n \geq 3$) normalized to the untreated control. *TPN*, TAP-binding protein tapasin; *TAP1/2*, transporter associated with antigen processing 1/2; *B2M*, β-2-microglobulin.

**Supplementary Table 1: Oligonucleotides used in qPCR experiments**

| Target Gene | Abbreviation | Primer name | Primer sequence |
|---|---|---|---|
| β-actin | *ACTB* | ACTB qPCR fw | GAAGCATTTGCGGTGGACGAT |
| | | ACTB qPCR rv | TCCTGTGGCATCCACGAAACT |
| $\beta_2$-microglobulin | *B2M* | B2M qPCR fw | CTCGCGCTACTCTCTCTT |
| | | B2M qPCR rv | AAGACCAGTCCTTGCTGA |
| E-cadherin | *CDH1* | CDH1 qPCR fw | TCCCTTCACAGCAGAACTAACA |
| | | CDH1 qPCR rv | AGTCACACACGCTGACCTCTAA |
| N-cadherin | *CDH2* | CDH2 qPCR fw | TGGGAATCCGACGAATGG |
| | | CDH2 qPCR rv | TGCAGATCGGACCGGATACT |
| claudin 1 | *CLDN1* | CLDN qPCR fw | CCTATGACCCCAGTCAATGC |
| | | CLDN qPCR rv | TCCCAGAAGGCAGAGAGAAG |
| glyceraldehyde-3-phosphate dehydrogenase | *GAPDH* | GAPDH qPCR fw | GGACTCATGACCACAGTCCAT |
| | | GAPDH qPCR rv | AGGTCCACCACTGACACGTT |
| metallo matrix protease 2 | *MMP2* | MMP2 qPCR fw | ATGGCTACCGCTGGTGCGG |
| | | MMP2 qPCR rv | GGTGCAGCTCTCATATTTGTTGCC |
| slug | *SNAI2* | SNAI2 qPCR fw | GACCCTGGTTGCTTCAAGGA |
| | | SNAI2 qPCR rv | TGTTGCAGTGAGGGCAAGAA |
| snail | *SNAI1* | SNAI1 qPCR fw | CATCCTTCTCACTGCCATG |
| | | SNAI1 qPCR rv | GTCTTCATCAAAGTCCTGTGG |
| tapasin | *TPN* | TPN qPCR fw | TGGGTAAGGGACATCTGCTC |
| | | TPN qPCR rv | ACCTGTCCTTGCAGGTATGG |
| transporter associated with antigen processing 1 | *TAP1* | TAP1 qPCR fw | GGAATCTCTGGCAAAGTCCA |
| | | TAP1 qPCR rv | TGGGTGAACTGCATCTGGTA |
| transporter associated with antigen processing 2 | *TAP2* | TAP2 qPCR fw | CCAAGACGTCTCCTTTGCAT |
| | | TAP2 qPCR rv | TTCATCCAGCAGCACCTGTC |
| transforming growth factor beta 1 | *TGFB1* | TGFB1 qPCR fw | GACTCGCCAGAGTGGTTATCTT |
| | | TGFB1 qPCR rv | CTGAAGCAATAGTTGGTGTCCA |
| transforming growth factor beta receptor 1 | *TGFBR1* | TGFBR1 qPCR fw | TGGCAGTAAGACATGATTCAGC |
| | | TGFBR1 qPCR rv | TAGATGTCAGCACGTTTGAAGG |
| transforming growth factor beta receptor 2 | *TGFBR2* | TGFBR2 qPCR fw | AGATACATGGCTCCAGAAGTCC |
| | | TGFBR2 qPCR rv | ACTTCTCCCACTGCATTACAGC |
| vimentin | *VIM* | VIM qPCR fw | GGAGATGCTTCAGAGAGA |
| | | VIM qPCR rv | TCTTCGTGGAGTTTCTTC |
| zinc finger and homeobox transcription factor-1 | *ZEB1* | ZEB1 qPCR fw | GCCAATAAGCAAACGATTCTG |
| | | ZEB1 qPCR rv | TTTGGCTGGATCACTTTCAAG |

Abbreviations: fw, forward; rv, reverse.

**Supplementary Table 2: Antibodies used for flow cytometry**

| molecule | antibody | company | isotype | fluorophore | order no. |
|---|---|---|---|---|---|
| HLA-ABC | Anti-HLA-ABC | Beckman Coulter | IgG2a | FITC | IM1838U |
| HLA-BC | Anti-human HLA BC | eBioscience | IgG1 | APC | 17-5935-42 |
| B7-H1 | Anti-human CD274 | eBioscience | IgG1 | PE | 12-5983-42 |
| B7-H2 | Anti-human B7RP-1 | eBioscience | IgG1 | PE | 12-5889-73 |
| B7-H3 | Anti-hB7-H3 | R&D systems | IgG1 | APC | FAB1027A |
| B7-H4 | Anti hB7-H4 | AbD serotec | IgG1 | FITC | MCA2632F |
| ICAM-1 | CD54-FITC | Beckman Coulter | IgG1 | FITC | IM0726U |
| TIM-3 | Anti-TIM-3 FITC | Miltenyi Biotec | IgG1 | FITC | 130-104-646 |
| isotype control | IgG2a(mouse)-FITC | Beckman Coulter | IgG2a | FITC | A12689 |
| isotype control | IgG1(mouse)-FITC | Beckman Coulter | IgG1 | FITC | A07795 |
| isotype control | IgG1(mouse)-PE | Beckman Coulter | IgG1 | PE | A07796 |
| isotype control | IgG1(mouse)-APC | eBioscience | IgG1 | APC | 400122 |
